# Postoperative radiotherapy for patients with completely resected stage IIIA‐N2 non‐small cell lung cancer: opt‐in or opt‐out

**DOI:** 10.1111/1759-7714.14335

**Published:** 2022-02-02

**Authors:** Lucheng Zhu, Bing Xia, Shenglin Ma

**Affiliations:** ^1^ Department of Radiotherapy Affiliated Hangzhou Cancer Hospital, Zhejiang University School of Medicine Hangzhou China; ^2^ Department of Oncology Affiliated Hangzhou Cancer Hospital, Zhejiang Chinese Medical University Hangzhou China

**Keywords:** adjuvant radiotherapy, lung cancer, IIIA‐N2 disease

## Abstract

The role of adjuvant radiotherapy in completely resected pIIIA‐N2 non‐small cell lung cancer (NSCLC) has long been debated. Evidence from previous retrospective and prospective studies showed that postoperative radiotherapy could reduce the incidence of local recurrence and prolong disease‐free survival, while two recently reported randomized controlled trials (lung ART and PORT‐C) both demonstrated no survival benefit of postoperative radiotherapy. The great gap between our knowledge and reality has made us rethink the value of postoperative radiotherapy. In this mini review, we elaborate on the role of postoperative radiotherapy in completely resected pIIIA‐N2 NSCLC.

## Graphical abstract

Non‐small cell lung cancer with N2 is a heterogeneous disease group. Postoperative radiotherapy is still necessary in the management of high‐risk pIIIA‐N2 patients.

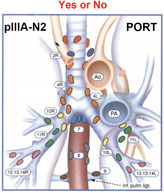

## INTRODUCTION

Locally advanced non‐small cell lung cancer (NSCLC) is thought to be the most challenging disease because of considerable heterogeneity and poor prognosis which requires multidisciplinary team management and effective multimodality combination. For those patients with completely resected IIIA‐N2 NSCLC, the role of postoperative radiotherapy (PORT) remains controversial. The 1998 PORT meta‐analysis showed a detrimental effect in patients with completely resected NSCLC but a slight increase in survival in pN2 disease, presumably due to the excessive late side effect of obsolete radiation technique.^1^ After that, SEER‐based analyses and several retrospective studies indicated that PORT using modern technologies reduced the local‐regional recurrence and conferred to the improvement of overall survival in patients with pN2 NSCLC.^2^ However, there has been a lack of prospective studies to validate the efficacy of modern radiation technique‐based PORT until the results of the Lung ART and PORT‐C which were recently reported (Table 1 summarizes the main findings of PORT studies).^7^, ^9^ Disappointingly, both trials showed that PORT reduced local‐regional recurrence but without survival benefit, and objections against PORT thus caused some confusion in clinical decision‐making.

**TABLE 1:** Comparison of main finding of different PORT studies

Study name	Study type	Published years	Number of patients	Disease stage	Main findings
PORT meta‐analysis^1^	Meta‐analysis	1998	2128	I–III	PORT increase risk of death in stage I/II disease The value of PORT in stage III/N2 was not clear
SEER^2^, ^3^	Retrospective	2006	7465	II–III	PORT was associated with better survival in patients with N2 nodal disease but not in patients with N0‐1 nodal disease
Wang^4^	Retrospective	2011	221	pIIIA‐N2	PORT significantly prolonged OS and DFS PORT prolonged locoregional recurrence‐free survival and distant metastasis‐free survival.
NCDB^5^	Retrospective	2015	4483	pN2	PORT was associated with better 5 year‐OS
Fu^6^	Retrospective	2021	1401	pIIIA‐N2	PORT significantly reduced the risk of LRR and improved OS in high‐risk population (Heavy cigarette smoking history, clinical N2 status, and the number of positive lymph nodes >4)
PORT‐C^7^	RCT	2021	394	pIIIA‐N2	PORT did not increase 3‐year DFS and OS PORT increased 3‐year DFS and but no OS in per‐protocol population
Lung ART^8^	RCT	2021	501	pN2	PORT did not increase 3‐year DFS OS data was not mature

Abbreviations: DFS, disease‐free survival; NCDB, National Cancer Data Base; OS, overall survival; PORT, postoperative radiotherapy; RCT, randomized‐controlled trial; SEER, Surveillance, Epidemiology, and End Results database.

It would be arbitrary to deny the value of PORT simply based on statistical results of one or two studies, and it is worth discussing and exploring the truth behind these numbers in more detail. The Lung ART trial was initiated in August 2007 with target accrual of 700 patients to show a 10% improvement of 3‐year disease free survival (DFS). Due to slow enrollment, a new target of 500 patients was adopted and the difference of 3‐year DFS was modified to 12% (42% in PORT arm and 30% in control arm). By the end of August 2018, 501 patients were enrolled into the trial, and preliminary results were reported in 2020 ESMO. In the ITT analysis, the primary endpoint was no significance between the two arms, the rate of 3‐year DFS was 47.1% and 43.8% in the PORT and control arms, respectively (HR = 0.85, *p*‐value = 0.16). Notably, over such a long enrollment period, the treatment of NSCLC has progressed including systemic and local therapy, especially with regard to radiation techniques. In the trial, 89% of the patients in the PORT arm received 3D‐CRT and it was difficult to achieve the optimal protection of normal tissue exposure compared to IMRT. Additionally, the Lung ART contouring protocol documented that the next nodal station should be included in the CTV when metastases were identified in a nodal station, and it was not difficult to guess that 1 and 2 stations were included in most cases, resulting in a relatively large radiation field.^10^ Therefore, it is easy to understand that cardiopulmonary toxicity caused up to 16% of deaths in the Lung ART trial.

In the RTOG 0617 study, multivariate analysis showed that heart V5 was an independent predictor of survival in patients with locally advanced NSCLC treated with concurrent chemoradiotherapy, and researchers and clinicians should therefore pay more attention to cardiopulmonary toxicities in these patients.^11^ A secondary analysis of RTOG 0617 was performed to compare IMRT with 3D‐CRT; of enrolled 482 patients, compared to 3D‐CRT cohort (53%), IMRT (47%) produced lower heart doses, less ≥grade 3 pneumonitis and better quality of life without local control sacrifice. In the Lung ART trial, the majority of the patients received 3D‐CRT based PORT and the median mean heart dose was 13.4 Gy. Atkins et al. analyzed 748 locally advanced NSCLC patients treated with thoracic radiotherapy (78% with 3D‐CRT) and found that the mean radiation dose delivered to the heart was associated with a significantly increased risk of major adverse cardiac events (adjusted HR: 1.05/Gy; *p* < 0.001) and all‐cause mortality (adjusted HR: 1.02/Gy; *p* = 0.007). Compared to the mean heart dose <10 Gy, >10 Gy would increase 34% hazard ratio of all‐cause mortality.^12^ Robert et al. reported similar findings, with a 2‐year cumulative incidence of grade ≥3 cardiac events of 18% in those with a mean heart dose above 11 Gy and 2% in those below 11 Gy.^13^ Presumably, excluding competing factors for tumor progression, there may be more adverse cardiopulmonary events with longer follow‐up which, to some extent, could offset the local benefit from PORT in Lung ART.

The actual rate of mediastinal relapse in the control arm was 46%, and PORT significantly reduced the rate to 25%, even in Lung ART ITT analysis (HR: 0.45, 0.30–0.69). As for the benefit of local control, this was not transformed into survival improvement, and it is worth further consideration. In addition, the Lung ART study was an international multicenter clinical trial conducted in five countries, and variations of target delineation and violations of radiotherapy quality assurance were also challenging that could potentially confound study results. Both RTOG 0617 and Proclaim trials showed that treatment at institutions with higher clinical trial accrual volume was associated with better clinical outcomes in LA‐NSCLC, highlighting the importance of ensuring radiation quality in RT clinical trials.^14^, ^15^

**FIGURE 1:**
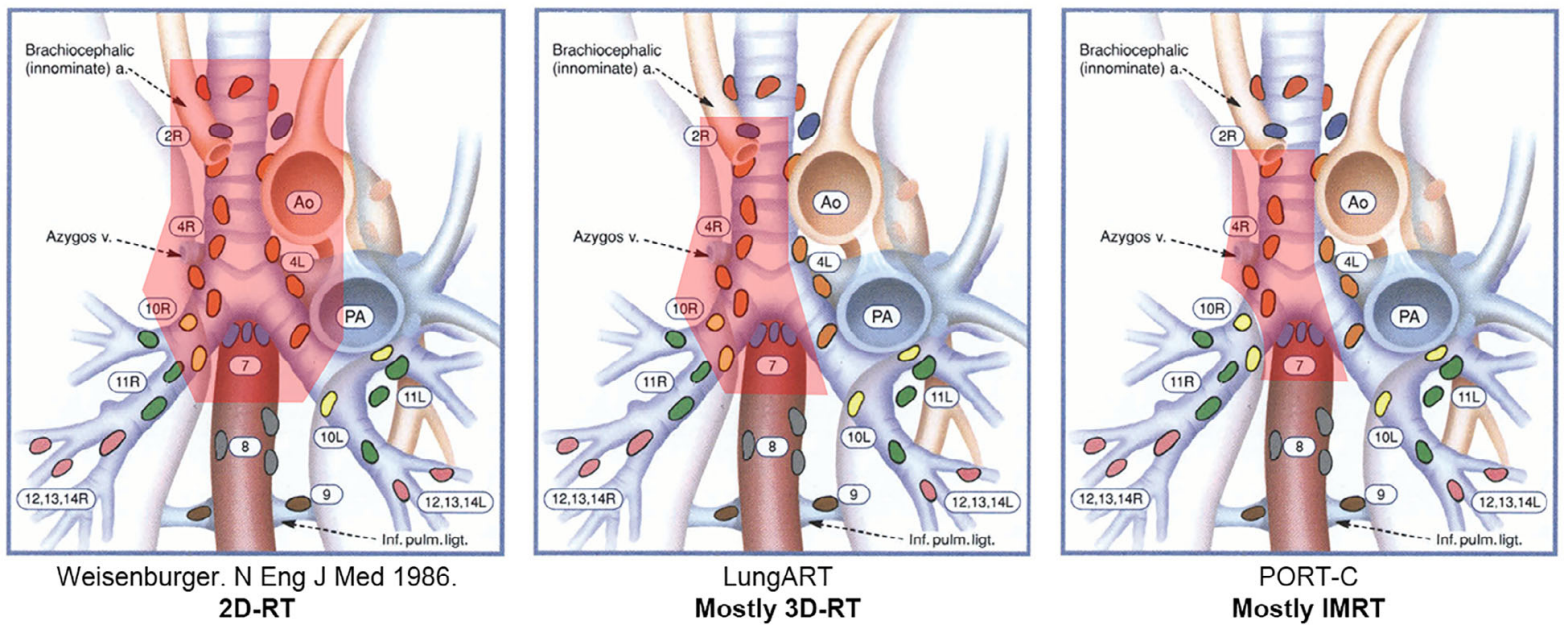
Comparison of radiotherapy target volumes between different studies in a right lung cancer patient with positive nodal stations 4R and 7 (Mountain‐Dresler) (left). The clinical target volume was superiorly by the suprasternal notch and inferiorly by a point 5 cm below the carina, the bronchial stump, ipsilateral hilum and vascular shadows of the mediastinum bilaterally (middle). The clinical target volume included the upper lymph node station (2R) and lower lymph node station (10R) to the involved lymph node regions as well as the bronchial stump and ipsilateral hilum (right). The clinical target volume included the bronchial stump, right hilum, and subcarinal, mediastinal lymph nodes (4R, 2R)

The PORT‐C study was also a phase III clinical trial conducted to evaluate the value of PORT in pN2 NSCLC with a target 3‐year DFS improvement of 14%, which enrolled 364 patients between January 2009 and December 2017 at a single cancer center in China. As shown in Figure 1, compared to Lung ART, a limited CTV (generally including ipsilateral hilum, subcarinal region, and ipsilateral mediastinum) and low prescribed dose of 50 Gy was used, and 89% of the patients were treated with IMRT that brought lower radiation dose in normal tissue. The actual median lung V20 and heart V30 were 16.7 and 10.4%, respectively (lung V20 and heart V30 were 23 and 15% in Lung ART), which resulted in low toxicity and good tolerability. Only three cardiopulmonary associated deaths were reported (3.1% in the whole population, estimated <6% in PORT group). In spite of this, PORT did not significantly improve the 3‐year DFS rate in a modified ITT population (40.5% vs. 32.7%; HR = 0.84, *p*‐value = 0.20). Poor adherence of the protocol in this study may account for no difference statistically; 44 of 184 patients (21.7%) in the PORT arm and 10 of 180 patients (5.6%) in the control arm refused scheduled treatment. Actually, in the sensitivity analysis, PORT significantly improved local control and DFS in the per‐protocol and as‐treated population. Therefore, PORT‐C should not simply be regarded as a negative study because of statistical underpower, and we should not easily negate the value of PORT in pN2 patients. Conversely, in a sense, this study identified that PORT reduces the risk of local recurrence and holds the promise of survival benefit.

Local tumor control is critical to achieve the goal of a radical cure for solid tumors. The treatment of breast cancer is the most successful model of comprehensive therapy, and PORT significantly improves local control and overall survival in both patients undergoing mastectomy and breast conserving‐surgery, indicating that early elimination of local tumor cells has the advantage of reducing subsequent metastasis and improving survival. In particular for early‐stage breast cancer, multiple randomized phase 3 studies designed to assess the role of adjuvant radiotherapy have demonstrated a remarkable risk reduction in local recurrence rates; however, no survival benefit was observed until the meta‐analysis of EBCTCG in which individual information in 23 500 participants was aggregated to evaluate the value of PORT and the survival benefit was finally demonstrated.^16^ In previous studies, patients with pN2 NSCLC had a poor prognosis, with a reported locoregional recurrence rate of 30%–60%.^17^, ^18^ Theoretically, local and survival advantage will be derived from PORT in this setting. However, both Lung ART and PORT‐C enrolled a limited number of patients; the former reduced the enrollment target, while the latter had a higher violation rate. Furthermore, DFS was used as the primary endpoint in the two trials, which was not an ideal parameter for evaluation of a local treatment; it was difficult to accurately reflect the value of PORT because of the competitive risk of distant metastases, particularly for pN2 population. If the effect of local control on overall survival is to be observed, a larger sample size and longer follow‐up may be needed, and the competing impact of distant metastasis should be reduced as far as possible.

Molecular targeted therapies and immunotherapies have improved outcomes markedly in advanced NSCLC, and the experience of successful treatment is also moving forward. The use of these agents in the adjuvant phase is also bound to affect the risk of both distant metastases and local recurrence. The ADAURA study showed that only 7% of the patients who received adjuvant osimertinib suffered locoregional‐only recurrence (18% in the placebo group), thereby suggesting that effective systemic therapy can lower the risk of local recurrence.^19^ However, the proportion of patients with N2 in this study was less than one third, and it was difficult to provide sufficient information about the impact of osimertinib on the local control in the *EGFR* mutation population. Recently, the results of immune checkpoint blockers (ICB) in resected NSCLC (IMPower 010) patients have been published in the Lancet.^20^ In the trial, patients with completely resected stage IB–IIIA NSCLC received up to four cycles of adjuvant chemotherapy, which was then randomized to atezolizumab maintenance treatment or best supportive care. The primary endpoint, DFS, showed a statistical difference in all of three predefined populations; for the patients with stage II–III disease, 3‐year DFS was 55.7 and 49.4% in the atezolizumab and control arms, respectively. Despite a moderate improvement in DFS outcome with adjuvant ICB, disease progression occurred in nearly half of patients, indicated that multiple therapeutic methods should be organically integrated to improve the overall therapeutic effect. Interestingly, in those patients with disease recurrence, more than half had locoregional failure irrespective of whether they received ICB or not (60.1% and 57.9%, respectively), suggesting that locoregional control will be of higher importance when systemic therapy is more effective. Evidence regarding radiation and immunotherapy response from the post‐hoc analysis of Keynote‐001 showed that significantly prolonged survival was observed in NSCLC patients who had previously received any radiotherapy compared with those who had not, indicating that the addition of radiotherapy can also improve the effect of ICB. With regard to the contribution of radiotherapy to locoregional control and its synergistic effect with ICB, the paradigm of combined adjuvant ICB and PORT is an area worth exploring. In addition, neoadjuvant ICB was also investigated widely in NSCLC and a high rate of major pathological response was observed. Undoubtedly, this will complicate the evaluation of postoperative pathological N2 and the value of PORT.

NSCLC with N2 is a heterogeneous disease group, including anatomical (location and number of involved nodes) and biological (histopathological and genetic) diversity,^21^ which may obscure the potential benefits of PORT. How to identify the patient subgroups who might derive the greatest benefit from PORT is therefore important for successful treatment. Unfortunately, so far, we have no reliable clinical or biological indicators to select an appropriate patient for PORT. In the literature, reported clinical factors for high local recurrence in pN2 NSCLC included heavy ex‐smokers, squamous cell carcinoma, ≥4 positive nodes or a high LNR (positive/resected lymph node ratio).^22^, ^23^ However, all these characteristics are derived from retrospective studies with limited sample size and lack of external validation. It remains unclear if the better outcome of these patients can be attributed to additional PORT based on these characteristics in a prospective manner. Future studies should take into account more detailed clinical features and molecular genetic information to accurately identify the appropriate patients who will benefit or not from PORT. Currently, minimal residual disease (MRD) is widely investigated in the study of systemic adjuvant therapy after radical treatment. Recently, He et al. found that longitudinal circulating tumor DNA (ctDNA) analysis was a promising tool for the detectection of MRD in NSCLC.^24^ Post surgical ctDNA positivity was significantly associated with worse recurrence‐free survival. Adjuvant therapy might be unnecessary in ctDNA‐negative patients with a minimal improvement in reducing their relapse risk. In this study, 46.6% (48/103) of patients had stage IIIA–IIIB disease. Locoregional relapse rates were 31.8% (7/22) and 12% (3/25) in MRD‐positive and ‐negative patients, respectively. Another prospective study also demonstrated MRD‐positive patients who received adjuvant therapies had improved relapse‐free survival (RFS), whereas MRD‐negative patients receiving adjuvant therapies had lower RFS.^25^ These data indicate the potential value of MRD on PORT. Therefore, how to integrate MRD in the management of pN2 NSCLC patients and perform adjuvant therapy according to the relapse risk deserves further investigation. In addition, more advanced imaging and radiation techniques, tighter dose restrictions and intense monitoring should be integrated into PORT as much as possible in order to minimize the risk of cardiopulmonary toxicity.

So now, what should we do in clinical practice when we encounter a patient with pN2 NSCLC? Recently, a survey was organized by the European Society for Radiotherapy and Oncology (ESTRO) reporting PORT in pN2 NSCLC patients investigated by 22 experts. After the presentation of the Lung ART trial, 82% of experts still used PORT for pN2 patients with risk factors in which the prominent consideration was extent of the lymph nodes (extracapsular nodal extension, bulky/multiple lymph nodes, multistation/level lymph nodes).^26^ Although PORT‐C did not show a statistically DFS improvement of PORT in this setting, no distinct survival disadvantage was provided by PORT based on the IMRT technique. Because there is a high locoregional recurrence risk in this population, PORT can be recommended to patients with good performance, ≥4 positive nodes or high LNR after a detailed multidisciplinary discussion. Also, the drugs used for systemic therapy should be seriously considered in order to avoid potential increased toxicity of combination therapy. The risks of cardiopulmonary toxicity also need to be taken into consideration.

## CONFLICT OF INTEREST

The authors confirm that there are no conflicts of interest.
